# Trends and Predictors of Pediatric Negative Appendectomy Rates: A Single-Centre Retrospective Study

**DOI:** 10.3390/children10050887

**Published:** 2023-05-15

**Authors:** Miro Jukić, Petra Nizeteo, Jakov Matas, Zenon Pogorelić

**Affiliations:** 1 Department of Surgery, School of Medicine, University of Split, Šoltanska 2, 21000 Split, Croatia; petra.nizeteo@gmail.com (P.N.); jmatas@mefst.hr (J.M.); zpogorelic@gmail.com (Z.P.); 2 Clinic of Pediatric Surgery, University Hospital of Split, Spinčićeva 1, 21000 Split, Croatia

**Keywords:** acute appendicitis, incidence, rate, negative appendectomy, children

## Abstract

Background: Appendectomy is still the standard treatment for acute appendicitis in the majority of centers. Despite all available diagnostic tools, the rates of negative appendectomies are still relatively high. This study aimed to determine negative appendectomy rates and to analyze the demographic and clinical data of the patients whose histopathology report was negative. Methods: All patients younger than 18 years who underwent appendectomy for suspected acute appendicitis in the period from 1 January 2012 to 31 December 2021 were included in the single-center retrospective study. Electronic records and archives of histopathology reports were reviewed for patients with negative appendectomy. The primary outcome of this study was a negative appendectomy rate. Secondary outcomes comprehended the rate of appendectomies and the association of age, sex, body mass index (BMI), values of laboratory markers, scoring systems, and ultrasound reports with negative histopathology reports. Results: During the study period, a total of 1646 appendectomies for suspected acute appendicitis were performed. In 244 patients, negative appendectomy was reported regarding the patients’ pathohistology. In 39 of 244 patients, other pathologies were found, of which ovarian pathology (torsion and cysts) torsion of greater omentum and Meckel’s diverticulitis were the most frequent. Finally, the ten-year negative appendectomy rate was 12.4% (205/1646). The median age was 12 years (interquartile range, IQR 9, 15). A slight female predominance was noted (52.5%). A significantly higher incidence of negative appendectomies was noted in girls, with a peak incidence between the ages of 10 and 15 years (*p* < 0.0001). Male children whose appendectomy was negative had significantly higher BMI values compared to female patients (*p* = 0.0004). The median values of white blood cell count, neutrophil count, and CRP in the patients with negative appendectomy were 10.4 × 10^9^/L, 75.9%, and 11 mg/dL, respectively. The median of Alvarado’s score was 6 (IQR 4; 7.5), while the median of the AIR score was 5 (IQR 4, 7). The rate of children with negative appendectomy who underwent ultrasound was 34.4% (84/244), among which 47 (55.95%) concluded negative reports. The rates of negative appendectomies were not homogenous in terms of distribution regarding the season. The incidence of negative appendectomies was more frequent during the cold period of the year (55.3% vs. 44.7%; *p* = 0.042). Conclusions: The majority of negative appendectomies were performed in children older than 9 years and most frequently in female children aged 10 to 15 years. In addition, female children have significantly lower BMI values compared to male children with negative appendectomy. An increase in the utilization of auxiliary diagnostic methods such as computed tomography could affect the reduction in the pediatric negative appendectomy rate.

## 1. Introduction

Acute inflammation of the appendix (acute appendicitis) is one of the most common causes of abdominal pain, which requires surgical attention, in children and adults, with an incidence of 1 in 1000 people per year [1,2]. The lifetime risk of developing the disease is slightly higher in male compared to female patients (8.6% vs. 6.7%), but females have a higher lifetime risk of undergoing appendectomy (23.1% vs. 12%) [1,2]. In children, the age-specific incidence progresses from extremely low in the newborn period to a peak in incidence between the ages of 12 and 17 [3]. The mortality rate is low, less than 1% [2]. Western countries are currently monitoring a stable incidence of appendicitis, while newly industrialized countries are recording an increase [4]. In addition, it has been shown that the incidence of acute appendicitis is lowest in winter and that air pollution and smoking, as well as a diet low in fiber and high in fat, are possible risk factors for the development of this disease [4].

Acute appendicitis is a clinical diagnosis based on a patient’s detailed history and patient examination. Typical clinical presentation is found in about 50% of patients. Other atypical forms of appendicitis are presented depending on the localization of the appendix or the patient’s age [5]. The most common symptom with the onset of pain is loss of appetite, nausea, and vomiting, a mild or moderate increase in body temperature (<38 °C), constipation, and diarrhea [5]. Symptoms of a retrocoecal appendix can include pain in the right lower back, while symptoms of a pelvic appendix can be pain in the groin, hematuria, and dysuria. Laboratory biomarkers are widely used for the diagnosis of acute appendicitis. Leukocytosis (>11 × 10^9^/L), the value of C-reactive proteins (CRP) (>8 mg/L) and neutrophilia are non-specific findings and have little diagnostic value, but combined, these parameters have a very high sensitivity for the diagnosis of acute appendicitis [6,7]. Recent studies clearly showed that several new biomarkers for acute appendicitis, such as hyperbilirubinemia, hyponatremia, hyperfibrinogenemia, pentraxin-3, neutrophil gelatinase-associated lipocalin, leucine-rich α-2-glycoprotein 1 (LRG1) or interleukin-6, showed good sensitivity and specificity for the detection of acute appendicitis. In addition, these markers also showed good predictive values for distinguishing between complicated and simple acute appendicitis [8,9,10,11,12,13,14,15]. A more recent study showed that salivary LGR1 may be a promising biomarker for the detection of acute appendicitis in pediatric patients [16].

If the clinical diagnosis is not clear, an abdominal ultrasound examination is recommended [17]. It is a non-invasive method that avoids radiation and is associated with a sensitivity of 71 to 94% and a specificity of 81 to 98%. It is reliable for determining the presence of inflammation of the appendix, but not for excluding it [17]. Abdominal computed tomography is superior to ultrasound in the accuracy of diagnosing appendicitis due to its high sensitivity of 76 to 100% and high specificity of 83 to 100% [17]. Magnetic resonance is recommended for pregnant women or children in whom ultrasound examination was not diagnostically useful [17]. To objectify and investigate suspected acute appendicitis diagnosis independently of the physician’s clinical experience, different scoring systems and scales were developed. The most frequently used scoring systems are Alvarado and AIR (Appendicitis Inflammatory Response) [18,19,20,21,22,23]. When diagnosing children with suspected acute appendicitis, the Alvarado, AIR, and PAS (Pediatric Appendicitis Score) score and the *pARC* (Pediatric Appendicitis Risk Calculator) are used [21]. The *pARC* is a new scoring system, compared to previous scores and it includes age, sex, duration of symptoms, pain when walking, coughing, or jumping, and the absolute number of neutrophils [22]. The AIR score and the pediatric risk calculator for appendicitis have significantly higher specificity and positive predictive values compared to the Alvarado and the PAS score [21,23].

The standard treatment for acute appendicitis is appendectomy. After removal of the appendix due to suspicion of acute appendicitis, it is recommended to send the specimen for pathohistological analysis [24]. One of the main reasons for this is the possibility of identifying malignancy in 1% of patients [24]. It is most often a neuroendocrine tumor of the appendix–carcinoid, adenocarcinoma, or mucinous cystadenoma [24]. When finding features of inflammation in an appendix, the pathologist should always describe their pattern [25]. Features that may indicate other processes of the appendix that are not only specific to primary acute appendicitis should also be taken into account [25]. If there are no inflammatory changes, i.e., a normal appendix is proven, the appendectomy is confirmed as negative [25]. In certain cases, there is no other clinically determinable cause for the typical symptoms of acute appendicitis [25]. It has been proposed that a change in the expression of cytokines or neurogenic hyperplasia could be an explanation for appendicular pain [25]. The incidence of negative appendectomy in the population varies worldwide, ranging from 1 to 40%, but in the majority of the reports, the incidence of negative appendectomies ranges from 10 to 15% and is proven to be significantly more likely to occur in females, rural hospitals, and those of a black racial background [24,26,27,28]. The acceptable rate is considerably higher in children, possibly due to the challenge of obtaining an accurate clinical history and physical examination of young patients [26].

This study aimed to investigate the incidence of negative appendectomies, as well as demographic and clinical characteristics with the separate use of diagnostic tools in children whose appendectomy was negative during the study period.

## 2. Materials and Methods

### 2.1. Patients

Out of 1680 children who underwent an emergency appendectomy, between 1 January 2012 and 31 December 2021, a total of 244 patients were included in this retrospective observational study. The inclusion criteria were as follows: all patients under the age of 18 who underwent appendectomy for suspected acute appendicitis and received a negative appendicitis histopathology report. A negative histopathology report implied that there were no inflammatory components in the appendix specimen. In opposition, patients over the age of 18, children with confirmed acute appendicitis in the histopathology reports, children with other appendiceal pathologies (parasitic infestation or neoplastic pathology), children who underwent incidental or elective appendectomy, and patients with incomplete data in case records were excluded from the study. Before conducting a data search, ethics approval was obtained from the Ethics Review Board of the University Hospital of Split (reference: 500-03/21-01/189; date of approval: 11 February 2022).

### 2.2. Outcomes of the Study

The primary outcome of this study was a negative appendectomy rate in children. Secondary outcomes comprehended the rate of appendectomies and the association of age, sex, body mass index (BMI), values of laboratory markers, scoring systems, and ultrasound reports with negative histopathology reports in children who underwent appendectomy for suspected acute appendicitis.

### 2.3. Data Collection and Study Design

**Figure 1:**
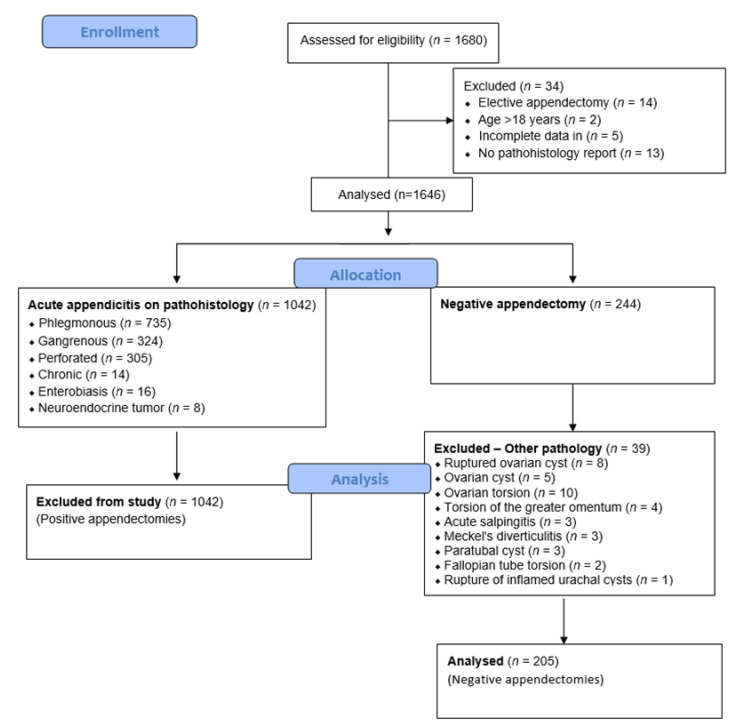
A flowchart diagram of the study.

After the medical records of all the children who underwent appendectomy for suspected acute appendicitis were reviewed, 34 children were excluded from the study because they met one or more of the exclusion criteria. Finally, histopathology reports of 1646 children were analyzed. A total of 244 patients were found to have negative appendectomy in the histopathology report and they were considered for further analysis. Demographic data (age, gender, and body mass index), preoperative laboratory values (leukocytes, neutrophil leukocytes, and CRP), preoperative abdominal ultrasound findings, and clinical findings (clinical examination, data on nausea/vomiting, loss of appetite, body temperature, and pain migration) were recorded. The pathohistological findings of the appendix in which no inflammatory component was described were interpreted as negative, while the findings of phlegmonous, gangrenous, chronic inflammation, enterobiasis, and carcinoid appendix were interpreted as positive pathohistological findings. The diagnosis of phlegmonous or suppurative acute appendicitis is established when neutrophil infiltration in the mucosa, submucosa, and muscle layer can be observed under the microscope, the inflammation is transmural, the ulcerations are extensive, intramural abscesses may be present and the thrombosis of blood vessels can be observed [25]. Necrotizing or gangrenous acute appendicitis is diagnosed when transmural inflammation, areas of necrosis and extensive mucosal ulceration are microscopically visible [25]. Chronic appendicitis is characterized by predominantly mononuclear inflammatory cell infiltrates with connective tissue present [25]. Appendectomies with negative pathohistological reports are defined as negative, and appendectomies with positive pathohistological reports as positive. The negative pathohistological reports were then divided into subgroups depending on the presence of some other pathological substrate that could have been the cause of the clinical presentation of acute appendicitis. The patients with negative appendectomies were subdivided into two-time frames regarding the year of surgery (2012–2017) and (2018–2022) to compare the main outcomes of the study. The patients were also divided by seasons based on their occurrence in the Northern Hemisphere. The flow chart of the study is shown in Figure 1.

### 2.4. Statistical Analysis

Statistical data analyses were performed using JASP (Jeffrey’s Amazing Statistics Program) version 0.16.2 (JASP Team, Amsterdam, The Netherlands) and Microsoft Excel (version 2013). The median (Md) and interquartile range (IQR) were used for quantitative variables. To describe the distribution of categorical variables of our study, relative and absolute frequencies were used. The normality of the distribution of numerical variables was tested by the Shapiro–Wilk test. Comparative analyses were performed using the chi-squared test for categorical variables and the t-test for independent samples for numerical values. *p* values < 0.05 were considered as significant.

## 3. Results

### 3.1. An Incidence of Negative Appendectomies

During the study period, a total of 1680 children were operated on because of acute appendicitis. Among the remaining subjects, 244 (14.8%) had negative appendectomy results. Out of 244 patients, in 39 of them, other pathologies were found, of which ovarian pathology (torsion and cysts) torsion of the greater omentum and Meckel’s diverticulitis were the most frequent. Finally, the ten-year negative appendectomy rate was 12.4% (205/1646). The incidence of appendectomies and negative appendectomies for each year are shown in Table 1. The incidence of negative appendectomies is stable with minor oscillations, which are not statistically significant (*p* < 0.455). When comparing the first five-year period with the last five-year study period, a slight decrease in negative appendectomy rates can be observed.

The demographic and clinical characteristics of subjects with negative appendectomy are shown in Table 2. In total, 128 (52.46%) female and 116 (47.54%) male children were included in the study. The median age of all participants was 12 years with an interquartile range of 9 to 15 years, which means that in our sample, 75% of the children were older than 9 years. The median result of the Alvarado scale was 6, while the median result of the AIR scale was 5. These results placed the majority of participants in the moderate- and medium-risk group.

Comparing the first five years with the second five years of the investigated period, no significant differences were found in regard to the demographic, laboratory, and clinical characteristics, except in the positive abdominal ultrasound examination (Table 3).

**Figure 2:**
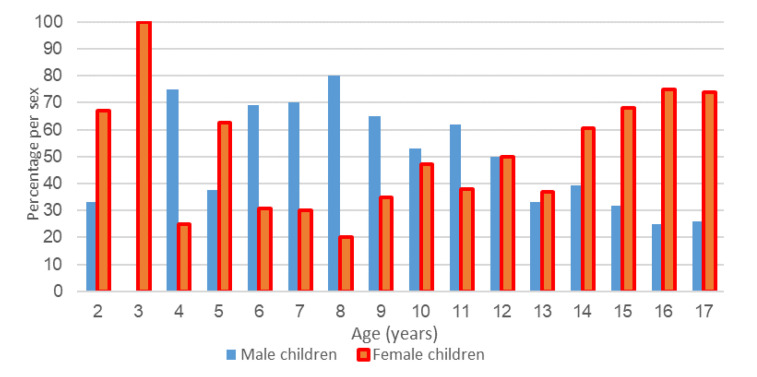
Distribution of subjects with negative appendectomies by age and sex.

The distribution of participants by gender and age is shown in Figure 2. The age range of subjects was from 2 to 17 years. The median age of male children was 10 (IQR 8; 13.25), while in female children, it was 14 (IQR 10; 15.75) years.

Comparing the values of the investigated parameters between boys and girls, statistically significantly older age was observed in girls with negative appendectomy, as well as a significantly lower median value of the BMI percentile in boys with negative appendectomy (Table 4). There is no correlation between sex and positive/negative ultrasound findings (*p* = 0.811).

**Figure 3:**
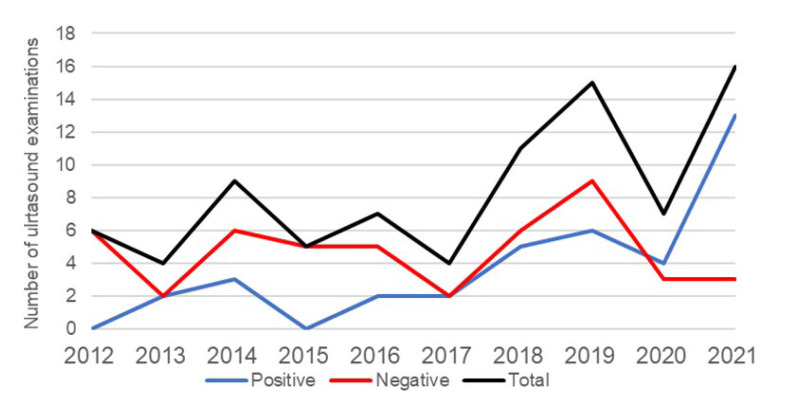
An incidence of performed preoperative ultrasounds in patients with negative appendectomy.

Male children with negative appendectomy had significantly higher BMI values than female patients (*p* = 0.0004), as stated in Table 4. Generally, among the patients, most were of healthy weight (134 (55%)), whilst the minority were underweight (3%), overweight (15%), and obese (27%). During the study period, an increase in the total number of performed ultrasound examinations, in patients with negative appendectomy results, was recorded (Figure 3). In 2012, 6 (20%) examinations were performed, while in 2021, 16 (69.6 %) examinations were performed, which is an increase of 3.48 times. At the beginning of the SARS-CoV-2 (COVID-19) pandemic, the number of examinations decreased, so in 2019, the number of examinations was 15 (57.7%), and in 2020, the number was 7 (41.1%). The highest number of negative abdominal ultrasound examinations was performed in 2019 with a frequency of nine (34.6%).

### 3.2. Analysis of Pathohistological Findings

Pathohistological findings were analyzed for each child with suspected acute appendicitis that was operated on during the investigated period and divided by the seasons as they occurred in the Northern Hemisphere. During the summertime, the incidence of positive appendectomies was the highest with an average of 31.3%, while the incidence of negative was the highest during the autumn with a percentage of 27.9%. The rates of negative appendectomies were not homogenous in terms of distribution regarding the season. The incidence of negative appendectomies was more frequent during the cold period of the year (55.3% vs. 44.7%; *p* = 0.042) (Table 5).

## 4. Discussion

When observing the incidence of negative appendectomy, as a quality measure of a surgical center, it should be taken into account that its definition is not harmonized by agreement in the scientific community. There have not been many systematic review articles or meta-analyses published so far on the incidence of negative appendectomy and the current knowledge is mainly based on research at the institutional or national level. Differences in definitions, criteria, and a large number of smaller studies are arguments that support the explanation of the large variation in the incidence of negative appendectomy reported in the world [26]. Some researchers define a negative appendectomy as one in which the appendix had a normal appearance at the time of surgery, while others use the final histopathological diagnosis for the definition [27,29].

Furthermore, in the histopathological diagnosis of appendicitis, its stage is also not agreed upon. Mariadason et al., in a retrospective study that covered a period of 15 years and included 1306 patients, proved a higher incidence of negative appendectomies after a change in the pathohistological criteria for the diagnosis of acute appendicitis [30]. In addition to the aforementioned definitions, it is important in the research methodology to emphasize the method of classifying subjects with a final diagnosis of parasitic infection or infestation, malignancy, or other primary pathologies of the appendix. Maloney et al. emphasized in their research that if only the number of normal appendix pathologies was taken, without the number of other primary pathologies found, the incidence of negative appendectomies would be 4.3% lower [26].

The criteria for calculating the incidence of negative appendectomies are described in detail in this study. Our results of the overall incidence are comparable and similar to the results of studies published so far on the incidence of negative appendectomies in children and adults [28,29,31]. Some authors doubted whether the laparoscopic approach increases the number of negative appendectomies. A recent study clearly showed that laparoscopic appendectomy should be offered as the method of choice in any patient population with suspicion of acute appendicitis and that the laparoscopic approach does not increase the number of negative appendectomies [31].

Recent studies have reported a drastic reduction in the incidence of negative appendectomies in children to 0 or 1% [32,33]. The reason for such a low incidence may be the consideration of only the intraoperative findings and setting the age limit of the subjects at more than 5 or less than 18 years of age. These two groups, children under 5 years of age and children younger than 18 years, which includes female children of reproductive age, are the risk groups for a negative appendectomy [34,35]. In addition, the increased use of ultrasound and CT has contributed to the decrease in incidence [34].

On the other hand, O’Sullivan et al. in their work reported a relatively high incidence of negative appendectomies in their institution, with a value of 31.9% [36]. Such a result is explained by the criteria for categorizing negative findings regarding the histopathology [36]. To investigate the predictive factors of negative appendectomy, Jeon et al. compared the demographic characteristics, leukocyte count, and radiological and clinical characteristics of patients with negative appendectomy and those with a confirmed diagnosis of acute appendicitis [37]. The results showed the following statistically significant independent predictive factors of negative appendectomy: age < 15 years, normal leukocyte count and 6 mm diameter of the appendix on CT. Normal values of neutrophils and CRP are not significantly related to negative appendectomy [37]. A study that included only a pediatric population also reported a normal leukocyte count as a predictive factor of a negative appendectomy [38]. In a retrospective study, Chiang et al. concluded that inflammatory markers such as leukocytes, neutrophils, and CRP used together have the best negative predictive value in confirming the diagnosis of acute appendicitis in children [39]. In our subjects, the median leukocyte count was within the normal reference values, which are in accordance with those studies [37,38,39]. In contrast to the mentioned studies, our results showed that the median of neutrophils and CRP was elevated [37,39].

A possible cause of the unexpected results is the smaller sample of subjects for those two laboratory markers. Research by Kaiser and colleagues showed a significant decrease in the incidence of negative appendectomies in children after the introduction of ultrasound and CT in the treatment of children with suspected acute appendicitis [40]. They point out an increase in performed ultrasound examinations in a nine-year period from 1% to 98% and an increase in the performed CT examinations from 0% to 59% as well, as a decrease in the incidence of negative appendectomies from 23% to 4% [40]. Radiological methods were not applied to a sufficient extent in our study in the investigated period to enable the calculation of their true usefulness in this study, but a significant increase in the application of abdominal ultrasound examinations was recorded in the observed group of children. We think that there is no rational justification for the routine use of CT due to the high doses of radiation and magnetic resonance and the high prices, in addition to the aggravating circumstances of performing imaging in an already overworked system.

Despite the increased use of diagnostic methods such as ultrasound and scoring systems, no statistically significant decrease in the incidence of negative appendectomies was recorded. Acute appendicitis is a common surgical disease in children, many symptoms are non-specific and the clinical presentation is not always typical. Therefore, detailed medical histories and a carefully performed clinical examination, as well as the experience of a pediatric surgeon, continue to form the backbone of deciding on the surgical procedure [41]. Studies on the incidence of negative appendectomies report different gender prevalence rates as well as different age groups in which negative appendectomies are more common. Oyetunji et al. pointed out that the incidence of negative appendectomies decreases with age and that it is higher in children younger than 5 years compared to older children [27]. They explain that such a conclusion stems from the fact that children under the age of 5 years often have an atypical clinical presentation of acute appendicitis and there is greater uncertainty when establishing a diagnosis [27]. In the same study, a statistically significantly higher proportion of girls with a negative appendectomy of 9.3% versus 5.1% of boys was demonstrated [27]. Adiss et al. reported the incidence of negative appendectomies in the general population for a large number of subjects and also reported a higher number of negative appendectomies in females [42].

However, the age group of children with the most negative appendectomies was between 10 and 18 years, while the proportion of cases in children younger than 5 years was lower [42]. During the COVID-19 pandemic, the incidence of negative appendectomies reduced, but the incidence of complicated appendicitis compared to the pre-pandemic period was significantly higher [43,44,45]. In our study, the majority of the patients were children older than 9 years, which corresponds to the age at which appendicitis is more frequent in the pediatric population. We have proven a statistically significantly higher number of negative appendectomies in the age group of girls from 10 to 15 years of age. The obtained results are consistent with the higher incidence of negative appendectomies in the female population, where a wide spectrum of gynecological pathologies can often be the cause of pain in the right lower quadrant and lead to surgery because of similar clinical presentation, including acute appendicitis. In our study, when analyzing all the histopathological findings, the presence of inflammation in the mucosa and submucosa, serosa, and transmural was not separately categorized.

All such findings are marked as positive. If in our research, when defining a negative appendectomy, only the histopathological findings of a normal appendix were taken into account, with no other non-inflammatory pathology found, the total incidence of negative appendectomies would be 12.4%. Among other intraoperatively detected pathologies confirmed by histopathological diagnostics, the largest number of negative appendectomies is caused by a gynecological substrate, most often ovarian cyst perforation or an ovarian cyst itself and ovarian torsion. The results of the research of Oyetunji and colleagues are in accordance with ours and they also mention ovarian cysts among the most common other primary diagnoses in the case of negative appendectomies [27].

In this study, the incidence of negative appendectomies during the 10-year period was stable with minor oscillations. The possible reasons for the variation in the incidence in the investigated period and the absence of a downward trend are probably due to a longer learning curve and the arrival of new and young pediatric surgeons who are on duty in the emergency department and are responsible for indicating surgical intervention when acute appendicitis is suspected.

Our research has several limitations. The data were collected retrospectively and were also collected from a single center. Among the collected findings, there is variability in the diagnosis, depending on the surgeon. Despite this, the researchers studied a very common pathology of the population, which, unfortunately, is not always easy to diagnose. It can be useful in directing further research on the same or similar topic.

## 5. Conclusions

The majority of negative appendectomies were found in children older than 9 years of age and significantly more often in females aged 10 to 15 years. In addition, female children have significantly lower BMI values compared to male children with negative appendectomies. The incidence of negative appendectomies was more frequent during the cold period of the year. Increasing the use of auxiliary diagnostic methods, as well as working with more experienced radiologists in performing pediatric ultrasounds, and defining new and adapted grading scales, could reduce the incidence of negative appendectomies in the future.

## Figures and Tables

**Table 1:** The incidence of negative appendectomies by years.

Year	Appendectomies		*p* *
	Total (*n* = 1646)	Negative (*n* = 205)	
2012	178	26 (12.7)	0.455
2013	174	19 (9.3)	
2014	181	27 (13.2)	
2015	176	14 (6.8)	
2016	149	27 (13.2)	
2017	165	21 (10.2)	
2018	164	22 (10.7)	
2019	172	16 (7.8)	
2020	143	15 (7.3)	
2021	144	18 (8.8)	

Data are shown as frequencies/*n* as the total number (percentages); * chi-squared test.

**Table 2:** Demographic, laboratory, and clinical parameters of the subjects.

Parameter	Value
Demographic characteristics	
Age (years)	12 (9, 15)
Gender	
Male	97 (47.3)
Female	108 (52.7)
BMI percentile range	75.4 (41.8, 95.6)
Laboratory parameters	
Leukocytes (×10^9^/L)	10.4 (7.9, 14)
Neutrophils (%)	75.9 (65.5, 83)
CRP (mg/L)	11 (1.7, 44.8)
Diagnostic scores	
Alvarado score	6 (4, 7.5)
AIR score	5 (4, 7)
Abdominal ultrasound	
Positive (*n* = 84)	37 (44)
Negative (*n* = 84)	47 (56)

Data are shown as median (IQR) or frequencies/*n* as the total number (percentages); BMI—body mass index, CRP—C-reactive protein, AIR—Appendicitis Inflammatory Response.

**Table 3:** Comparison of five-year periods with regard to demographics, laboratory, and clinical parameters of subjects with negative appendectomy.

Parameter	2012–2016 (*n* = 107)	2017–2022 (*n* = 98)	*p*
Age (years)	13 (9, 15)	11 (8.3, 14)	0.144 ^†^
Male	56 (52.3)	41 (41.8)	0.729 *
BMI percentile range	78.1 (45.8, 97.3)	71.7 (37, 94)	0.120 ^†^
Leukocytes (×10^9^/L)	10.6 (7.6, 14)	10.1 (8.2, 14.2)	0.830 ^†^
Neutrophils (%)	76 (63.6, 84)	75.6 (66.9, 82.6)	0.733 ^†^
CRP (mg/L)	13.2 (1.7, 46.6)	8.7 (1.2, 35)	0.495 ^†^
Alvarado score	6 (4, 7)	6 (4, 8)	0.733 ^†^
AIR score	5 (4, 7)	5 (4, 6)	0.411 ^†^
Positive abdominal US	7 (22.6)	30 (56.6)	<0.0001 *

Data are shown as median (IQR) or frequencies/*n* as the total number (percentages); * chi-squared test; ^†^ t-test for independent samples; BMI—body mass index, CRP—C-reactive protein, AIR—Appendicitis Inflammatory Response, US—ultrasound.

**Table 4:** Comparison of demographic, laboratory, and clinical parameters of subjects with negative appendectomies.

Parameter	Male Children	Female Children	*p* ^†^
Age (years)	10.5 (8, 13.5)	14 (10, 15.8)	<0.0001
BMI percentile range	86.8 (45.4, 98.0)	64.7 (31.1, 89.4)	0.0004
Leukocytes (×10^9^/L)	11.4 (8.6, 14.5)	9.9 (7.5, 13.2)	0.118
Neutrophils (%)	79 (71.3, 86.9)	73.2 (62.7, 89.5)	0.062
CRP (mg/L)	17.1 (5.2, 55.3)	6.2 (1.2, 33.2)	0.138
Alvarado score	7 (4, 8)	6 (4, 7)	0.108
AIR score	5 (4, 7)	5 (3, 6)	0.038

Data are shown as median (IQR) or frequencies/*n* as the total number (percentages); ^†^ t-test for independent samples; BMI—body mass index, CRP—C-reactive protein, AIR—Appendicitis Inflammatory Response.

**Table 5:** Pathohistological findings in patients who underwent appendectomy due to suspected acute appendicitis.

Season	Pathohistological Finding		p *
	Positive (*n* = 1402)	Negative (*n* = 244)	
Winter	310 (22.1)	67 (27.4)	0.042
Spring	322 (22.9)	50 (20.5)	
Summer	439 (31.3)	59 (24.2)	
Autumn	331 (23.7)	68 (27.9)	

Data are presented as numbers (percentages); * chi-squared test.

## Data Availability

The data presented in this study are available upon request from the respective author. Due to the protection of personal data, the data are not publicly available.
